# Attention Cueing in Rivalry: Insights from Pupillometry

**DOI:** 10.1523/ENEURO.0497-21.2022

**Published:** 2022-06-22

**Authors:** Miriam Acquafredda, Paola Binda, Claudia Lunghi

**Affiliations:** 1 Department of Neuroscience, Psychology, Pharmacology and Child Health, University of Florence, Florence 50135, Italy; 2 Department of Translational Research and New Technologies in Medicine and Surgery, University of Pisa, Pisa 56126, Italy; 3 Laboratoire des Systèmes Perceptifs, Département d’Études Cognitives, École Normale Supérieure, Paris Sciences et Lettres University, Centre National de la Recherche Scientifique, Paris 75005, France

**Keywords:** attention, binocular rivalry, interocular grouping, pupillary light response, pupillometry, visual awareness

## Abstract

We used pupillometry to evaluate the effects of attention cueing on perceptual bi-stability, as reported by adult human observers. Perceptual alternations and pupil diameter were measured during two forms of rivalry, generated by presenting a white and a black disk to the two eyes (binocular rivalry) or splitting the disks between eyes (interocular grouping rivalry). In line with previous studies, we found that subtle pupil size modulations (∼0.05 mm) tracked alternations between exclusive dominance phases of the black or white disk. These pupil responses were larger for perceptually stronger stimuli: presented to the dominant eye or with physically higher luminance contrast. However, cueing of endogenous attention to one of the rivaling percepts did not affect pupil modulations during exclusive dominance phases. This was observed despite the reliable effects of endogenous attention on perceptual dominance, which shifted in favor of the cued percept by ∼10%. The results were comparable for binocular and interocular grouping rivalry. Cueing only had a marginal modulatory effect on pupil size during mixed percepts in binocular rivalry. This may suggest that, rather than acting by modulating perceptual strength, endogenous attention primarily acts during periods of unresolved competition, which is compatible with attention being automatically directed to the rivaling stimuli during periods of exclusive dominance and thereby sustaining perceptual alternations.

## Significance Statement

Binocular rivalry depends on attention. When it is diverted away from the stimuli, perceptual alternations slow down; when it is preferentially directed to one stimulus, perception lingers more on it, consistent with attention enhancing the effective strength of the rivaling stimuli. Here, we introduce pupillometry as a means to indirectly track changes in effective stimulus strength. We find that pupil size accurately tracks perceived luminance during two forms of rivalry: binocular rivalry and interocular grouping rivalry. Both show robust effects of attention cueing on perceptual dominance, but pupil modulations during exclusive dominance are unaffected by cueing. This suggests that endogenous attention does not affect perceptual strength during exclusive dominance, although it might do so during transition phases.

## Introduction

When stimuli in the two eyes are incompatible, binocular fusion fails and perception alternates between the monocular images (binocular rivalry; Wheatstone, 1838; Alais and Blake, 2015). Rivalry has been shown to depend on attention, as perceptual alternations tend to cease when attention is diverted away. When this happens, neural oscillations in early visual areas are also suppressed (Zhang et al., 2011), consistent with the notion that attention modulates the strength of early neural representations (Carrasco, 2011). These effects are adequately modelled by assuming that attentional resources are automatically driven to the dominant stimulus, unless engaged elsewhere; and that attention provides recurrent excitation of the corresponding monocular input, acting synergically with interocular inhibition to maintain the competition between eyes (Li et al., 2017). Besides diverting attention away from the stimuli, cueing attention to one of the rivaling stimuli can affect binocular rivalry, shifting perceptual dominance in favor of the cued percept (Meng and Tong, 2004; Mitchell et al., 2004; Chong et al., 2005; van Ee et al., 2006; Hancock and Andrews, 2007; Paffen and Alais, 2011; Dieter et al., 2016). However, the neural underpinnings of cueing effects have been less systematically studied and, to the best of our knowledge, no previous study has tested whether attention cueing affects the strength of early visual representations during rivalry.

Interocular competition can sometimes be overcome by pattern-based competition, as in interocular grouping rivalry (Alais et al., 2000), where monocular stimuli are complementary, e.g., two half gratings, and perception alternates between images grouped across eyes (Alais et al., 2000). The role of attention in interocular grouping rivalry has not been investigated. In general, attention cueing has more pronounced effects on more complex types of bistable perception, such as Necker cube or bistable structure from motion (Meng and Tong, 2004; van Ee et al., 2005), which could predict stronger attentional modulations in interocular grouping than in binocular rivalry.

Here, we propose pupillometry as a method to indirectly index the strength of competing visual representations and objectively quantify the effects of attention cueing on binocular and interocular grouping rivalry.

Pupil size is mainly set by retinal illumination through a simple subcortical circuit (Loewenfeld, 1993). However, light responses are modulated by saliency, attention, brightness illusions and contextual processing (Laeng et al., 2012; Binda and Murray, 2015; Wang and Munoz, 2015; Mathôt, 2018) indicating that the subcortical circuit is fed with cortical signals (Binda and Gamlin, 2017) that represent effective stimulus strength. As long as stimuli are tagged with different luminance, pupil diameter can be used to accurately and precisely track attention in space (Binda et al., 2013; Mathôt et al., 2013; Naber et al., 2013) and perceptual alternations over time (Lowe and Ogle, 1966; Einhäuser et al., 2008; Fahle et al., 2011; Naber et al., 2011; Turi et al., 2018; Tortelli et al., 2021b). Here, we exploited this strategy and used luminance to tag pupil responses to stimuli rivaling in perception (for an alternative approach that did not rely on luminance tagging, see Brascamp et al., 2021) . We predicted that, if attention cueing enhances the effective strength of the dominant stimulus, pupil modulations should be amplified. The amplification would provide an objective and time-resolved index of how attention affects binocular and interocular grouping rivalry.

## Materials and Methods

### Participants

We recruited 38 participants (17 males and 21 females including two authors, mean age 26.5 ± 0.69 years). Sample size was based on a power analysis that determined the minimum number of participants required to detect a medium sized effect (effect size 0.50, two tailed α 0.05, power 0.8 = 33 participants; we recruited a few more anticipating data losses that fortunately did not occur). Ten additional participants were recruited for the control experiment (nine females and one male, mean age 27.1 ± 0.82 years).

All participants had normal or corrected-to-normal visual acuity (ETDRS charts), normal stereopsis (TNO test), and normal color vision (Ishihara plates); balanced ocular dominance (excluding participants with ocular dominance higher than 70%); no self-reported history of eye surgery, other active eye diseases or mental illness.

### Ethics statement

The experimental protocol was approved by the local ethics committee (Comité d’Éthique de la Recherche de l’Université Paris Descartes, CER-PD:2019–16-LUNGHI) and was performed in accordance with the Declaration of Helsinki (DoH-Oct2008). All participants gave written informed consent and were reimbursed for their time at a rate of 10€/h.

### Apparatus, stimuli, and procedures

Experiments took place in a dark and quiet room. Visual stimuli were developed in MATLAB (The MathWorks Inc.) using Psychtoolbox-3 (Brainard, 1997) running on a PC (Alienware Aurora R8) and a NVIDIA graphics card (GeForce RTX2080). Visual stimuli were displayed on a 53.5-cm-wide monitor, driven at a resolution of 1920 × 1080 pixels. The display was linearized by γ-correction; it was seen through a four-mirror stereoscope which enabled dichoptic viewing of two display areas of 12 × 8° each; a chin rest was used to stabilize head position at 57 cm from the display. In each display area, a central red fixation point (0.15° in diameter) surrounded by a square frame (3.5 × 3.5°) were shown against a uniform gray background (luminance: 152 cd/m^2^).

The mirrors were carefully adjusted at the beginning of each session to ensure accurate alignment of the dichoptically presented squares. Participants were asked to keep their gaze on the fixation point shown at screen center and to refrain from blinking while the stimuli were on.

Dichoptic presentations consisted of two sets of stimuli, designed to elicit two forms of rivalry: binocular rivalry and interocular grouping rivalry.

**Figure 1.**
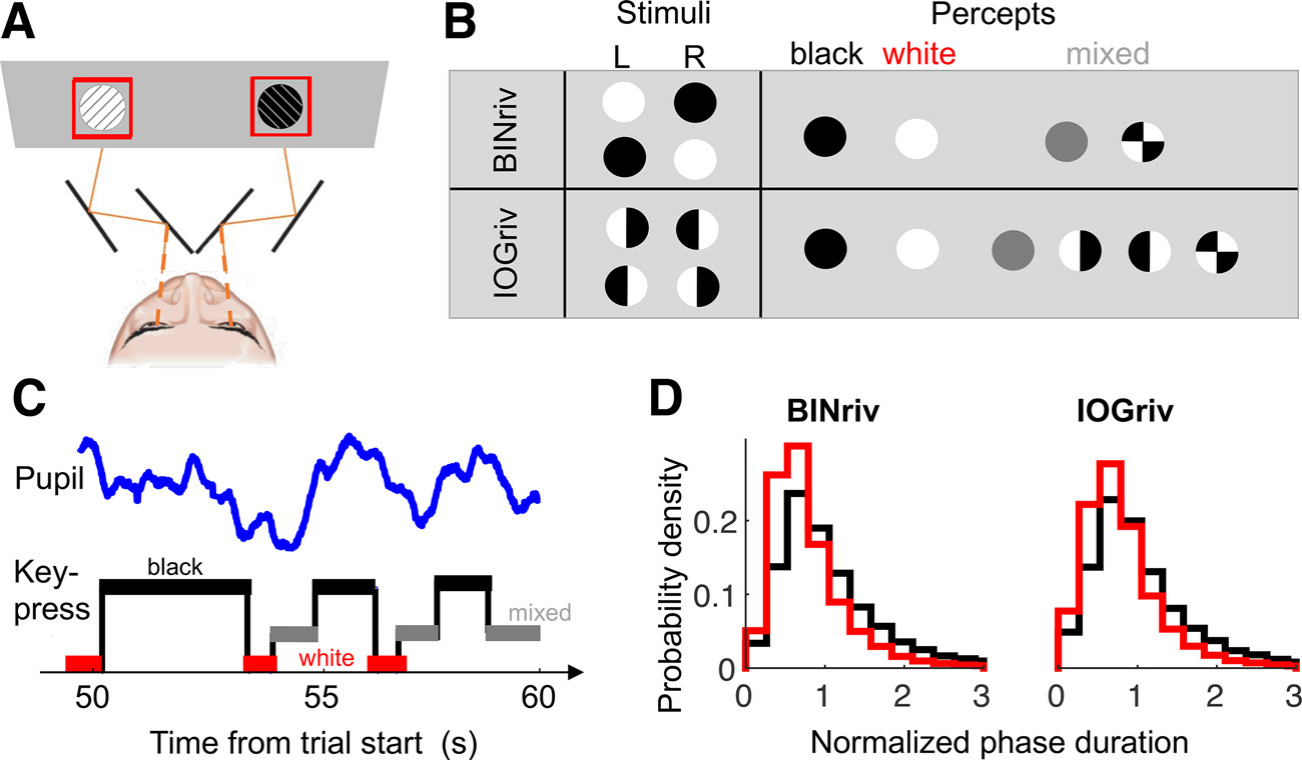
Dichoptic stimulation and rivalry dynamics. ***A***, Schematics of the stimuli (white or black patches overlayed with orthogonal thin lines), presented dichoptically through a four-mirror stereoscope. ***B***, Schematic representation of the possible stimulus configurations (thin lines omitted) and perceptual outcomes for binocular rivalry (BINriv) and interocular grouping rivalry (IOGriv). ***C***, Example traces from a segment of the experiment, where participants used keypresses to report the dominant percept (square wave) and we recorded pupil size modulations (blue wave). ***D***, Probability density function of the normalized phase durations for exclusive dominance of white or black disk percepts in binocular rivalry and interocular grouping rivalry.

For binocular rivalry, visual stimuli consisted of two disks (Fig. 1*A*), 3° in diameter, one white (maximum screen luminance 295 cd/m^2^) and one black (minimum screen luminance 10 cd/m^2^). Given the mid-level gray background, the two stimuli had virtually identical Weber contrast of 0.9, but they differed in terms of Michelson (0.3 for the white disk and 0.9 for the black disk). Perception alternated between exclusive dominance of the white and the black disk, or mixed percepts (either piecemeal or fusion). To discourage fusion, the disks were overlaid with thin orthogonal gray lines (45° clockwise or counterclockwise, one pixel wide, corresponding to 0.033°, and 0.5° apart, with the same luminance as the background).

For interocular grouping rivalry, the same stimuli (white/black disks with thin lines) were split vertically, and the two halves shown to the two eyes (Fig. 1*B*). Possible percepts were exclusive dominance of the white or the black disk (grouped interocularly), monocular percepts (half white half black disks, as shown to the left or to the right eye) and fusion or piecemeal percepts.

Stimuli were presented continuously for 3-min-long trials. Trials were separated by 60-s-long pauses with only the fixation point shown against the background. During this time, participants reported their perception of afterimages; analyses of this behavior will be reported in a separate publication. On each trial, a different combination of disk color and line orientation was presented to each eye; combinations varied pseudo-randomly across trials. Participants continuously reported perception by keeping one of three keys pressed: right or left arrows to report dominance of the stimulus with clockwise or counterclockwise tilted lines, or the down-arrow key any time dominance of either stimulus was incomplete (i.e., monocular percepts in interocular grouping rivalry, piecemeal and fusion events were not distinguished in our paradigm). We did not exclude data from any trial or participant. We eliminated perceptual phases shorter than 0.3 s (accounting for a total of 1.3% and 0.6% of recording time for binocular rivalry and interocular grouping rivalry, respectively), which we assumed to reflect keypress-errors or very fast switches or return transitions that would not be adequately tracked by the slow dynamics of the pupil. In total, we analyzed ∼500 perceptual phases per participant and stimulus type (591.23 ± 25.71 for binocular rivalry and 501.78 ± 23.97 for interocular grouping rivalry, mean ± 1 SE across participants).

Dominance phase distribution were adequately captured by a typical γ distribution (Levelt, 1967), with shape *α* and scale *β* parameters for binocular rivalry: *α =* 2.62; *β = 0.32* and for interocular grouping rivalry: *α =* 2.50 and *β = 0.34* in Equation 1. The goodness-of-fit (coefficient of determination *R*^2^) was 0.94 for binocular rivalry and 0.97 for interocular grouping:

(1) $$f\left(x|\alpha ,\beta \right)=\frac{1}{\beta^{\alpha }\Gamma (\alpha )}x^{\alpha -1}e^{\frac{-x}{\beta }}\text{ for }x,\alpha ,\beta >0.$$

Where Γ is the γ function and x the number of dominance phases.

Binocular rivalry and interocular grouping rivalry were both tested in three conditions: no attentional cue, black percept cued or white percept cued. In the latter, a “D” or a “B” letter was displayed at the beginning of the trial, cueing participants to endogenously focus their attention on the black or white disk throughout the rivalrous alternations.

We applied a fully randomized factorial design, where each participant completed 32 trials, divided into eight sessions of four trials each, one for each combination of rivalry type and attentional cueing, distributed over two days.

We also set-up a simulated rivalry stimulus, where a single white, black or half white and half black disk (the latter simulating mixed percepts) was shown monocularly to one eye (right or left in separate trials), alternating with phases of 2.5 ± 0.01 s. Four trials of this stimulus were always run at the beginning of the experiment, to help participants familiarize with the task.

Note that this is different from a standard “replay rivalry” as it involved a standardized alternation between stimuli, and it was merely intended to measure pupil modulations produced by the physical alternation of luminance stimuli.

### Eye tracking data acquisition and analysis

During rivalry and simulated rivalry, we monitored pupil diameter and two-dimensional eye position with an infrared camera (EyeLink 1000 system, SR Research) mounted below the monitor screen and behind the stereoscope. EyeLink data were streamed to the main computer through the EyeLink toolbox for MATLAB (Cornelissen et al., 2002) and thereby synchronized with participant’s keypresses. Pupil diameter measurements were transformed from pixels to millimeters using an artificial 4-mm pupil positioned at the approximate location of the participant’s eye.

Pupil and gaze tracking data consisted of 180 × 1000 (180 s at 1000 Hz) time points. These included signal losses, eyeblinks and other artifacts, which we cleaned out by means of the following steps (all implemented with in-house MATLAB software):

- Identification and removal of large artifacts: removal of time points with unrealistically small or large pupil size (>1.5 mm from the median of the trial or <0.2 mm, corresponding to blinks or other signal losses).

- Identification and removal of finer artifacts: identification of samples where pupil size varied at unrealistically high speeds (>10 mm/s, beyond the physiological range).

- Removal of low-frequency oscillations by subtracting a high-pass Butterworth filter with a threshold frequency of 0.1 Hz from each 180-s-long trial.

After this cleaning procedure was applied, we verified fixation stability by measuring the dispersion of eye position samples around the mean of each trial as the bivariate confidence ellipse area (BCEA), defined as the following:

(2) $$BCEA=2*k*\sigma_{H}*\sigma_{V}*{\left(1-\rho \right)}^{0.5}.$$

Where *k* is the confidence limit for the ellipse, *σH* and *σV* are the SD of eye positions in the horizontal and vertical meridian, respectively, ρ is the product-moment correlation of these two position components and *k *=* *1.14, implying that the ellipse included 68% (1-e^(-^*^k^*^)^) of the distribution. To test for possible differences in eye-movement patterns, we averaged the BCEA values across trials and entered these values in a 2 × 2 repeated measure ANOVA with factors: cueing condition (no cue/cueing) and rivalry type (binocular/interocular grouping rivalry). No main effect or interaction was significant, suggesting that fixation was equally stable across all conditions and rivalry types (main effect of cueing condition: *F*_(1,37)_ = 0.41, *p* = 0.52, logBF = –0.64; main effect of stimulus type: *F*_(1,37)_ = 0.44, *p* = 0.51, logBF = –0.69; cueing condition by stimulus type interaction: *F*_(1,37)_ = 0.70, *p* = 0.41, logBF = –0.54).

**Figure 2.**
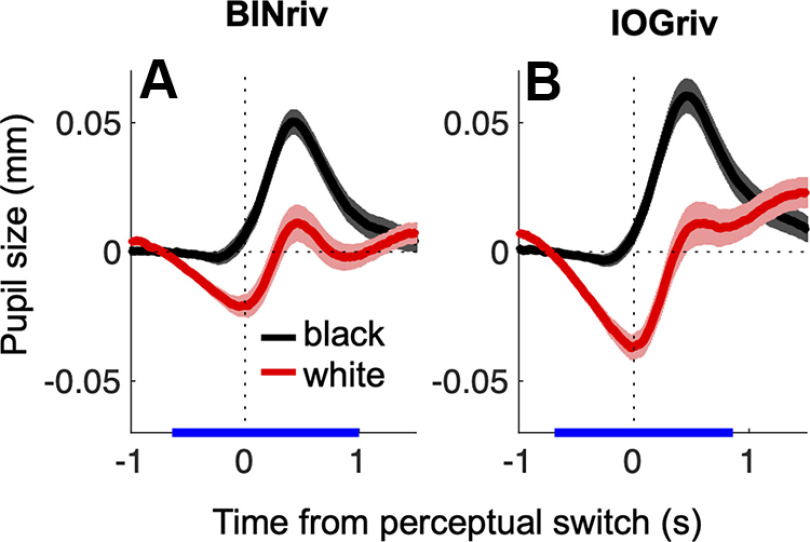
Pupil modulations track perceptual alternations. Baseline subtracted pupil size traces aligned to perceptual switches toward exclusive dominance of a white disk or a black disk percept and averaged across phases, separately for binocular rivalry (***A***) and interocular grouping rivalry (***B***). In all panels, shadings report mean ± 1 SE across participants and the blue marks on the *x*-axis highlight time points where pairwise comparisons between traces are significant (one tailed *t* test, *p* < 0.05 FDR corrected). Observations regarding the latency of the pupillary response and its relative magnitude are reported in Extended Data Figure 2-1, where nonbaseline subtracted traces are shown.

After cleaning, pupil data and continuous recordings of perceptual reports were down-sampled to 100 Hz, by taking the median of the retained time points in nonoverlapping time windows. If no retained sample was present in a window, that window was set to “NaN” (MATLAB code for “not a number”). Down-sampled pupil traces (to which we re-applied the second step of the cleaning procedure) were finally parsed into epochs locked to each perceptual switch (when the participant changed perceptual report) and labeled according to the color of the dominant stimulus after the switch. Pupil time courses were averaged across epochs for each participant; further averaging across participants yields traces in Figure 2. In order to minimize the impact of pupil size changes unrelated to the perceptual switches, we also analyzed data after subtracting a baseline from each epoch, measured in the −1:−0.5 s interval preceding the switch. To compare pupil size across dominance phases, stimuli and attention conditions, we extracted a pupil size index by averaging baseline corrected pupil size in the −0.5:1 s interval around the switch. Note that shifting the intervals for pupil baseline, or skipping the baseline correction step, affected the size of pupil modulations but it did not change our conclusion on the effects of attention and stimulus type (see Extended Data Fig. 4-1); also, we verified that attention cueing did not affect pupil baseline measures (see Extended Data Fig. 4-1).

10.1523/ENEURO.0497-21.2022.f2-1 Extended Data Figure 2-1 Pupil size modulations in rivalry and simulation conditions. Time course of the difference between pupil size during black and white percepts, computed in individual participants, and then averaged for binocular rivalry (dark blue curve), interocular grouping rivalry (light blue curve) and simulation (green curve). In all panels, shadings report mean ±1 SE across participants and the blue marks on the *x*-axis highlight time points where each trace is significantly different from 0 (one tailed *t* test, *p* < 0.05 FDR corrected). Pupil modulations during binocular and interocular grouping rivalry averaged, respectively, 39.14 ± 8.15% (mean ± 1 SE across participants) and 43.80 ± 10.26% of the pupil size modulations observed during simulated rivalry, as previously reported by Binda and colleagues (Binda et al.,2013) in a different spatial-attention task. Moreover, they consistently started before the perceptual switch, and this was more pronounced in the rivalry conditions than in the simulated rivalry (significant pupil difference started 160 ms before the switch in binocular and 320 ms in interocular grouping rivalry, compared with almost no latency for simulated rivalry). This finding, in line with Fahle et al. (2011) and Naber et al. (2011), may reflect the graded nature of rivalry transitions, which may delay change detection in rivalry compared with the sharp transitions used in the simulated rivalry condition. Download Figure 2-1, TIF file.

10.1523/ENEURO.0497-21.2022.f4-1 Extended Data Figure 4-1 Pupil modulations track perceptual alternations comparably across cueing conditions irrespectively of whether and how pupil traces are baseline corrected. Pupil size traces aligned to perceptual switches towards exclusive dominance of a white disk or a black disk percept and averaged across phases, separately for binocular rivalry (***A***) and interocular grouping rivalry (***B***) and separately for the two cueing conditions: cueing the white percept (dashed lines) or the black percept (continuous lines). Shading report ±1 SE across participants. These traces are computed without subtracting any baseline correction (***A***, ***B***) and after subtracting a baseline computed as average pupil size in the [–5:5] s interval around perceptual switch (***C***, ***D***). Note how the resulting traces are virtually indistinguishable: in both cases pupil size still allows to discriminate white and black percepts (red and black curves are clearly separated) but shows no effect of cueing (dashed and continuous lines are virtually superimposed, together with the blue traces). Coherently, we found that cueing did not affect preswitch pupil baseline used for the main figures, which was computed in the [–1:–0.5] s interval from the perceptual switch (main effect of cued percept: *F*_(1,37)_ = 1.51, *p* = 0.23, logBF = –0.60; dominant percept × cued percept interaction: *F*_(1,37)_ = 0.14, *p* = 0.71, logBF = –0.74). Download Figure 4-1, TIF file.

To quantify the effect of attention on both perceptual reports and pupil measurements, we computed indices of attentional modulation (AMI) for comparing perceptual and pupil measures in cueing versus in no-cueing trials. Specifically, we used Equations 3, 4, where PROP is the total dominance time of the white and black disk percepts divided by total testing time and PUPDIFF is the average pupil size difference between black and white disk dominance phases:

(3) $$\begin{array}{l} AMI_{prop}=\left(PROPpercept1_{cued}-PROPpercept2_{uncued}\right)-\\ \left(PROPpercept1_{nocue}-PROPpercept2_{nocue}\right) \end{array}$$

(4) $$AMI_{pup}=PUPDIFF_{cued}-PUPDIFF_{nocue}.$$

For the sake of clarity, we chose to quantify perceptual reports using dominance proportions; however, the same conclusions could be drawn analyzing mean phase durations instead.

To check for possible differences in the reliability of pupillary modulations across conditions, we also evaluated the cross-correlation between pupil size and perceptual reports. Previous studies reported that synchronization with rapid cognitive and perceptual events is more precise for pupil change rate (the first derivative of pupil size) than for pupil size (de Gee et al., 2020; Brascamp et al., 2021; Murphy et al., 2021), presumably because of the long temporal impulse-response function of the pupil, which results in a broad autocorrelation of this measure. In line with these studies, we opted to measure the cross-correlation between perceptual reports and the pupil-size change rate. For each participant, we averaged the normalized cross-correlation function across trials and stimulus type. We fit it with a Gaussian function (constrained to peak at lags smaller than 1.5 s and with SD smaller than 0.2 s) and compared its peak amplitude across conditions.

### Statistical approach

Significance was evaluated using both *p*-values and log-transformed JZS Bayes factors computed with the default scale factor of 0.707 (Wagenmakers et al., 2012). The Bayes factor is the ratio of the likelihood of the two models H1/H0 given the observed data, where H1 is the experimental hypothesis (effect present) and H0 is the null hypothesis (effect absent). A base 10 logarithm of the Bayes factor (logBF) larger than |0.5| corresponds to a likelihood ratio larger than 3 in favor of either H1 (when logBF > 0.5) or H0 (when logBF < −0.5) and this value is conventionally used to indicate substantial evidence in favor of either hypothesis (Kass and Raftery, 1995; Keysers et al., 2020). Bayesian ANOVAs were run in JASP, and the corresponding Bayes factors represent the change from before posterior inclusion odds (BFinclusion) computed across matched models. Moreover, following the review reviewed by Richardson (2011), we report partial η squares (η_p_^2^, computed in JASP) as effect size estimates for all factors in our repeated measures ANOVAs.

We estimated the internal consistency of our parameter estimates by split-half reliability. Each parameter was estimated twice per participant, on half the dataset (odd and even trials) and we evaluated the correlation of the two sets across participants. Finally, we evaluated the significance of behavioral attentional effects at the single participant level with a bootstrapping approach, by resampling (10,000 times, with reinsertion) dominance phases in cueing and no-cueing conditions, applying Equation 3, computing the proportion of samples where the attentional modulation index was larger than 0 or smaller than 0 and assigning the significance for *p* < 0.025.

### Control experiment

A control experiment was performed after the end of the study, with the aim of estimating the sensitivity of pupil size measurements to manipulations of stimulus strength. The original set-up was inaccessible at the time of testing, and we replicated the conditions of the main experiment as closely as possible in another set-up, using the same eye-tracker (EyeLink 1000 system, SR Research), similar mirror stereoscope and a computer that ensured equal performance. Specifically, stimuli were generated with the PsychoPhysics Toolbox routines (Brainard, 1997) and MATLAB (MATLAB r2010a, The MathWorks Inc.) housed in a Mac Pro 4.1, and displayed on a 52.5-cm-wide LCD screen with maximum screen luminance of 108 cd/m^2^. Instead of using the maximum and minimum screen output, we reduced luminance levels by about a factor of 10 to allow for modulations of stimulus contrast. We set the background luminance to 15 cd/m^2^ gray and tested six conditions: a no cue and white cued condition in which stimuli where 28 and 2 cd/m^2^ for the white and black disk, respectively, and four conditions where the Michelson contrast of the white disk stimulus was increased by 25%, 50%, 100% and 150% (luminance values: 33, 40, 63, and 108 cd/m^2^). Each condition was tested in four trials, and all data were collected over a single session.

## Results

We analyzed perceptual alternations and the associated pupil modulations during binocular and interocular grouping rivalry, in two conditions: with and without attentional cueing.

In no cueing conditions, pupil diameter reliably tracked perceptual alternations between a white and a black disk presented dichoptically, either one disk per eye generating binocular rivalry, or each disk split vertically between eyes generating interocular grouping rivalry (Fig. 1). Despite constant stimulation (hence constant luminance), pupils were relatively dilated when participants reported seeing black, compared with when they reported seeing white (Fig. 2*A*,*B*, black vs red line), in both types of rivalry.

**Figure 3.**
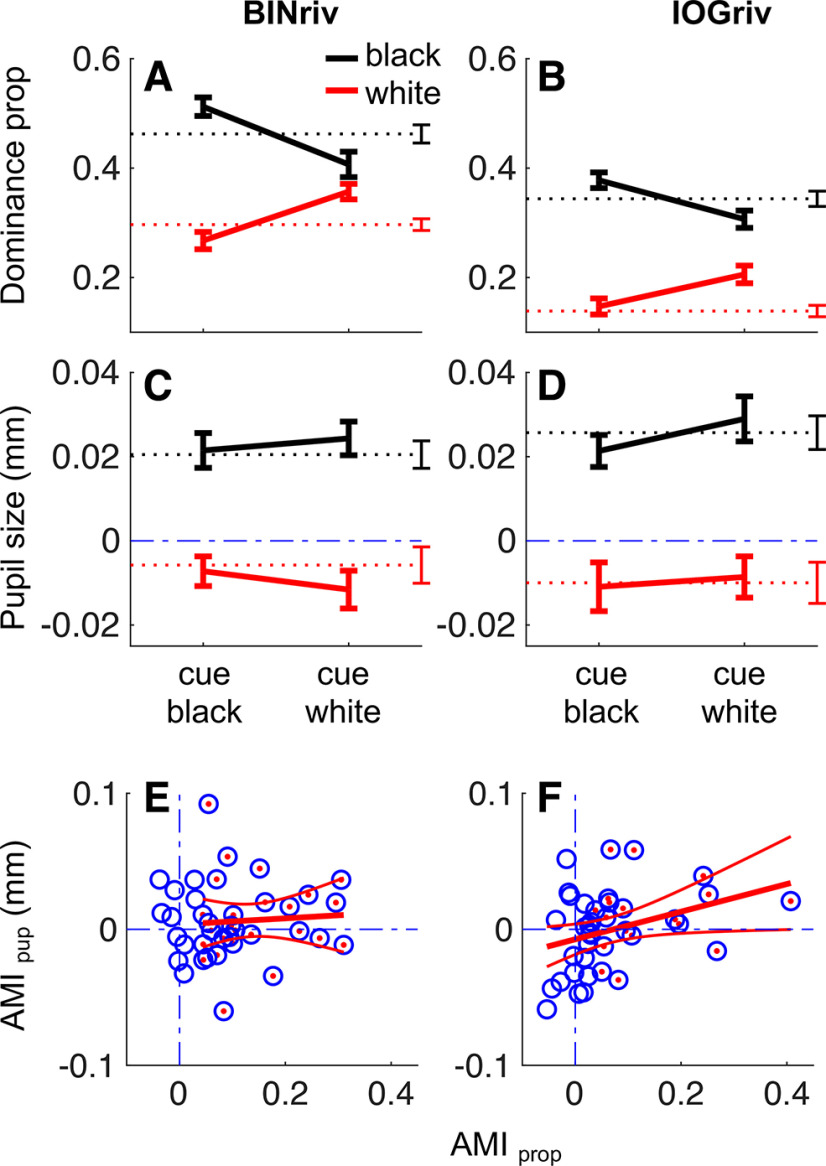
Attention cueing affect perceptual alternations but not pupil modulations. ***A***, ***B***, Perceptual dominance for exclusive white or black disk percepts, without attentional cueing (dashed lines) or when the white or the black disk percept were cued (continuous lines, cueing condition indicated on the abscissa). Error bars report ±1 SE across participants. ***C***, ***D***, Baseline corrected average pupil size computed in a fixed temporal window (between –0.5 and 1 s from the perceptual transition) during phases of exclusive dominance of the black (black line) and the white disk (red line). Results from the no-cueing condition are reported by dashed lines. Continuous lines report the results from the trials where the white or the black percepts were cued (separated on the abscissa). Error bars report ±1 SE across participants. ***E***, ***F***, Individual participants’ attentional modulation indices for perceptual dominance (*x*-axis) and pupil size (*y*-axis), computed with Equations 3, 4. Dash-dot blue lines mark the *x* = 0 and *y* = 0 lines, indicating no effect of attention cueing. Each circle reports results from one participant; red dots highlight participants with a significant attentional modulation index for perceptual dominance. Red lines show the best fitting line and its 95% confidence intervals. In all panels, the left column reports results for binocular rivalry and the right for interocular grouping rivalry.

The analysis of behavioral reports in the no cueing condition (Fig. 3, dotted lines) showed a net predominance of black disk percepts with respect to the white ones (binocular rivalry: *t*_(37)_ = 8.70, *p* < 0.001, logBF = 7.77; interocular grouping rivalry: *t*_(37)_ = 12.81, *p* < 0.001, logBF = 12.27). In line with the modified Levelt’s propositions and results by Qiu et al. (2020), this can be explained by the higher Michelson contrast of the black disk stimulus (recall that the background was set to mid-gray, resulting in identical Weber contrast but different Michelson contrast for the two disk stimuli). However, dominance of the black percept cannot logically explain the pupil modulations; moreover, while both black dominance and pupil size modulations varied across participants, the two were reliably uncorrelated (binocular rivalry: *r* = –0.09, *p* = 0.58, logBF = –0.83, interocular grouping rivalry: *r* = –0.20, *p* = 0.22, logBF = –0.58).

Figure 3*A*,*B* also show that exclusive percepts were much rarer in interocular grouping rivalry compared with binocular rivalry (*t*_(37)_ = 12.23, *p* < 0.001, logBF = 11.68), reflecting the response mapping we used; for interocular grouping rivalry, mixed reports included epochs where the individual monocular images in the left and right eye dominated.

Having established pupil size as a marker of perceptual dominance in both binocular and interocular grouping rivalry, we proceeded to assess the impact of attention cueing on perceptual alternations and pupil size modulations (Fig. 3, continuous lines).

As expected, perceptual dominance of the cued stimulus was enhanced, resulting in a significant interaction between dominant percept (white/black) and cued percept (cueing white/cueing black) on the proportion of exclusive dominance phases (Fig. 3*A*,*B*; Table 1, middle column). We summarized the effect of attention with an attentional modulation index (Eq. 3 in Materials and Methods), which was in the order of 10% for both types of rivalry (single participant data are shown on the abscissas of Fig. 3*E*,*F*). The effect was statistically reliable at the group level and, in most cases, at the individual participant level (bootstrapped attentional modulation indices were significantly higher than zero in 27/38 or 20/38 participants for binocular and interocular grouping rivalry, respectively, highlighted with a red dot in Fig. 3*E*,*F*; it was significantly lower than zero in only 2/38 participants for interocular grouping rivalry and in no participant for binocular rivalry). Attentional modulation indices were correlated between binocular rivalry and interocular grouping (*r* = 0.61, *p* < 0.001, logBF = 2.63), suggesting that they measure a relatively stable feature of our participants. In line with this, we found no indication that interocular grouping rivalry was more affected by attention than binocular rivalry; if anything, there was a small effect in the opposite direction (binocular rivalry minus interocular grouping rivalry, *t*_(37)_ = 2.33, *p* = 0.02, logBF = 0.28).

**Table 1:** The effects of cueing on proportions and pupil size

	Proportions	Pupil size
Dominant percept	*F*_(1,37)_ = 146.46* p < 0.001 logBF = 30.54 η_p_^2^ = 0.80	*F*_(1,37)_ = 46.09* p < 0.001 logBF = 22.52 η_p_^2^ = 0.55
		
Rivalry type	*F*_(1,37)_ = 82.62* p < 0.001 logBF = 25.49 η_p_^2^ = 0.69	*F*_(1,37)_ = 0.12 *p* = 0.73 logBF = −0.90 η_p_^2^ = 0.003
		
Cued percept	*F*_(1,37)_ = 5.37* p = 0.03 logBF = −0.82 η_p_^2^ = 0.13	*F*_(1,37)_ = 1.91 *p* = 0.17 logBF = −0.78 η_p_^2^ = 0.05
		
Dominant percept × rivalry type	*F*_(1,37)_ = 0.99 *p* = 0.33 logBF = −0.62 η_p_^2^ = 0.03	*F*_(1,37)_ = 0.17 *p* = 0.68 logBF = −0.72 η_p_^2^ = 0.005
		
Dominant percept × cued percept	*F*_(1,37)_ = 32.96* p < 0.001 logBF = 11.33 η_p_^2^ = 0.47	*F*_(1,37)_ = 1.78 *p* = 0.19 logBF = −0.53 η_p_^2^ = 0.05
		
Rivalry type × cued percept	*F*_(1,37)_ = 0.08 *p* = 0.77 logBF = −0.78 η_p_^2^ = 0.002	*F*_(1,37)_ = 1.92 *p* = 0.17 logBF = −0.54 η_p_^2^ = 0.05
		
Dominant percept × rivalry type × cued percept	*F*_(1,37)_ = 5.45* p = 0.02 logBF = −0.11 η_p_^2^ = 0.13	*F*_(1,37)_ = 0.03 *p* = 0.85 logBF = −0.41 η_p_^2^ < 0.001

Three-way ANOVA for attention cueing results, with factors: dominant percept (white/black disk), cueing (white/black cued), rivalry type (binocular/interocular grouping rivalry). These results were not affected by shifting the baseline or skipping this step (Extended Data Table 1-1). * for *p* < 0.05 or lower.

Based on the assumption that attention cueing boosts perceptual dominance by enhancing the effective strength of the cued percept, we expected to find an enhancement of the pupil responses accompanying perceptual alternations. For example, we predicted that dilations concurrent with black percept dominance would be increased when cueing black. To test for this effect, Figure 3*C*,*D* plots the mean baseline-corrected pupil size over a fixed interval [−0.5:1s] around the perceptual switch for each percept type and attention cueing condition (the same interval used for computing pupil size in the no-cue condition, shown by dotted lines). Red curves show pupil size during white percepts and black curves during black percepts; the two cueing conditions (white/black percept cued) are separated on the *x*-axis. The format is the same as that used to expose the effects of attention on the proportions of dominant percepts in Figure 3*A*,*B*. According to our hypothesis, attention should have affected mean pupil size, displacing the continuous curves away from the dashed lines that reports the no-cueing results. However, no such systematic displacement was observed. This was confirmed statistically (Table 1, rightmost column, both the main effect of cued percept and the interaction between dominant percept and cued percept are nonsignificant, with log Bayes factors < −0.5).

**Figure 4.**
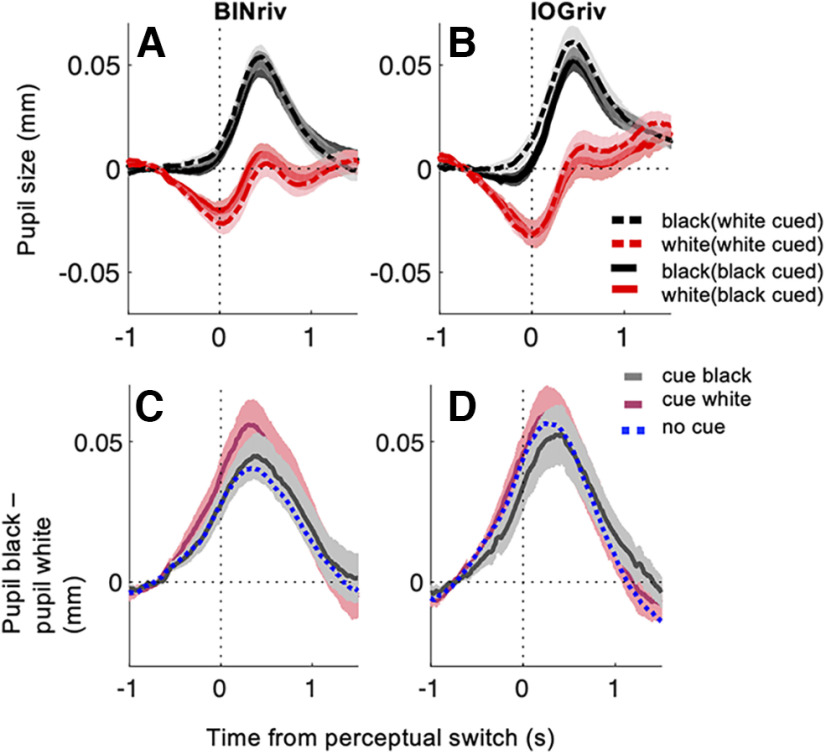
Pupil time courses are comparable across cueing conditions. ***A***, ***B***, Pupil size traces aligned to perceptual switches toward exclusive dominance of a white disk or a black disk percept computed across phases in individual participants and then averaged for each cueing condition, separately for binocular rivalry and interocular grouping rivalry. ***C***, ***D***, Time course of the difference between baseline corrected pupil size during black and white percepts, computed in individual participants and then averaged for each cueing condition. The resulting traces show no effect of cueing. The same conclusions can be drawn skipping the baseline correction step or defining pupil baseline over a wider temporal interval around perceptual switch, as shown in Extended Data Figure 4-1. In all panels, shadings report mean ±1 SE across participants.

Figure 4 shows the full pupil time courses across cueing conditions, using the same format as in Figure 1, and supporting the same conclusions drawn from Figure 3 and Table 1. The curves are remarkably similar regardless of whether the white or the black percept was cued; as a result, the amplitude of the pupil modulation (computed as the difference between pupil size during black–white disk percepts) was not affected by attention cueing. These figures were computed after baseline correcting pupil traces, i.e., subtracting the average pupil diameter preceding each perceptual switch before averaging traces across switches. We verified that our pupil baseline values were not affected by attention cueing and we checked that skipping this baseline correction step or defining pupil baseline over a wider temporal interval (−5:5 s, the whole interval over which we tracked pupil size for each perceptual switch) did not alter our conclusions (Extended Data Table 1-1; Extended Data Fig. 4-1).

We computed an attentional modulation index for pupil size (Eq. 4), logically similar to the attentional modulation index computed for perceptual dominance (Eq. 3). Attentional modulation indices for pupil size were distributed around zero for all participants (as shown on the ordinates of Fig. 3*E*,*F*). Even selecting the subsample of participants who showed a significant behavioral effect of attention cueing (Fig. 3*E*,*F*, red dots), the pupil attentional modulation remained nonsignificantly different from zero (*t*_(26)_ = 1.15, *p* = 0.25, logBF = –0.42 for binocular rivalry and *t*_(19)_ = 1.72, *p* = 0.10, logBF = –0.09 for interocular grouping rivalry).

Thus, our results show disagreement between pupillometric and behavioral measures of perceptual dominance. Only perceptual alternations were affected by attention cueing, not the accompanying pupil modulations. Could this be because of lack of sensitivity of pupillometry? We gathered several pieces of evidence against this possibility.

First, not only we did not find any evidence of an effect of attention, but we also obtained evidence in support of the null hypothesis (no effect of attention) using the Bayes factor (Keysers et al., 2020). Because the log-Bayes factor is defined as the log-likelihood ratio of the experimental and the null hypothesis, a value smaller than −0.5 indicates that the null hypothesis is three times more likely than the experimental hypothesis given the data (conversely, a log-Bayes factor larger than 0.5 implies that the experimental hypothesis is three times more likely than the null hypothesis given the data). As shown in Table 1, the effect of attention was associated with nonsignificant *p*-values mostly accompanied by log-Bayes factors smaller than −0.5, indicating substantial evidence (Kass and Raftery, 1995) against the hypothesis that attention cueing affects pupil size.

Second, we verified that the reliability of pupil measurements was high (test-retest reliability on the pupil size difference: *r* = 0.72, *p* < 0.001, logBF = 4.78 and *r* = 0.81, *p* < 0.001, logBF = 7.11 for binocular rivalry and interocular grouping rivalry, respectively), comparable to the reliability of the behavioral measurements (test-retest reliability for dominance proportions: *r* = 0.72, *p* < 0.001, logBF = 4.84 and *r* = 0.79, *p* < 0.001, logBF = 6.73 for binocular rivalry and interocular grouping rivalry, respectively).

Third, we found that pupil measurements were sensitive enough to report the slight unbalances between eyes observed in our set of (nonamblyopic) participants. This was shown by splitting the same set of perceptual phases (binocular rivalry with attention cueing) in two ways: according to whether the reported percept was cued or un-cued, and according to whether it matched the stimulus presented in the dominant or nondominant eye. This measure confirmed that pupil size was insensitive to attention cueing (pupil modulations were not different when the cued or un-cued stimulus was perceived, *t*_(37)_ = 1.23, *p* = 0.23, logBF = –0.46), but it did report eye-dominance (pupil modulations being larger in phases where percepts matched the stimulus in the dominant eye, *t*_(37)_ = 2.59, *p* = 0.01, logBF = 0.51).

Fourth, we ruled out the possibility that the amplitude of pupil modulations was already saturated in the no cue condition, by measuring pupil modulations in a simulated rivalry condition. The latter was not intended as a replay-rivalry condition; it was run at the beginning of the experiment, training our participants to report perceptual alternations, and it did not reproduce the dynamics of exclusive dominance and mixed percepts observed during rivalry. However, it did allow us to estimate the pupil modulations elicited by physical alternations of the white and black disk, which measures the maximum modulation possible elicited by perceptual alternations during rivalry. We found that the latter were ∼40% of the modulations during simulated rivalry (39.13 ± 8.15 for binocular rivalry and 43.80 ± 10.26 for interocular grouping rivalry, mean ± 1 SE across participants). This implies that there was ample space for the putative boosting effect of attention, ruling out ceiling as an explanation for the lack of such effect.

**Figure 5.**
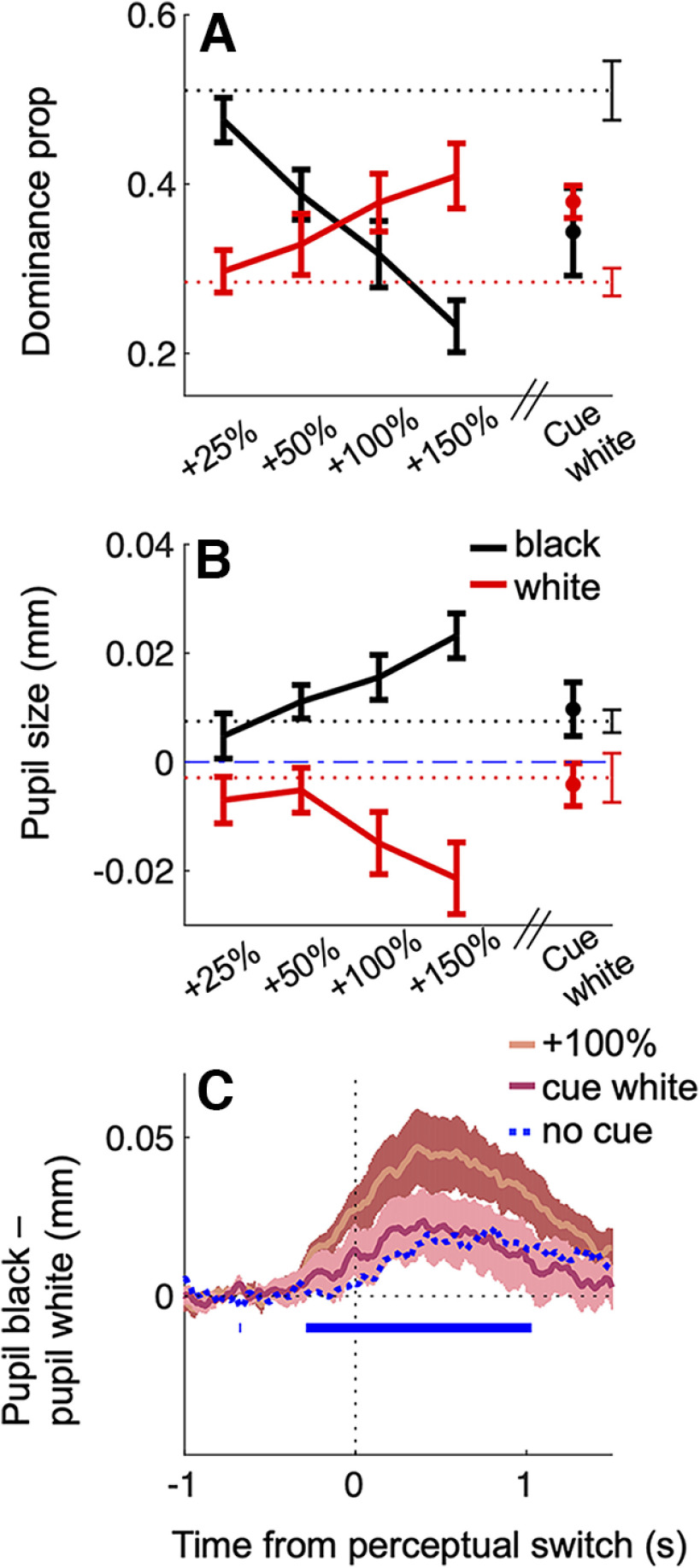
Effects of attention cueing versus enhancing contrast. ***A***, ***B***, Perceptual dominance for exclusive white or black disk percepts, in the no cue condition (dashed lines) or when the physical contrast of the white disk was enhanced/cued (continuous lines, contrast, or cueing condition indicated on the abscissa). Error bars report ±1 SE across participants. ***C***, Time course of the difference between baseline corrected pupil size during black and white percepts, computed in individual participants and then averaged for each condition. The blue marks on the *x*-axis highlight time points where pairwise comparisons between the +100% and the no cue condition traces are significant (*p* < 0.05 FDR corrected). Shadings report mean ±1 SE across participants.

Fifth, and finally, we showed with a control experiment that pupil size modulations reliably track changes in stimulus strength of a size compatible with those simulating the effects of attention cueing on perceptual dominance (Fig. 5). This was tested in a separate cohort of participants and with a different set-up, where we repeated the attentional manipulation (comparing trials where the white disk was cued vs no cue trials) and, in separate no cue trials, we manipulated the physical strength of the white disk increasing it by 25%, 50%, 100%, or 150% (for technical reasons, we had to decrease the average luminance of the stimuli in the equal contrast conditions, which was ∼10 times lower than in the main experiment).

A 2 × 5 repeated measures ANOVA showed that perceptual dominance was affected by the contrast modulation (Fig. 5*A*), resulting in a significant interaction between dominant percept (white/black) and contrast condition on the proportion of exclusive dominance phases (Table 2). Importantly, the same pattern was found for the pupil modulation (Fig. 5*B*). Increasing the difference in the physical strength between the two simuli (i.e., increasing the contrast of the white disk), enhanced the pupil response, resulting in a significant interaction between the pupil response to the dominant percept and contrast condition.

**Table 2:** Contrast enhancement effects

	Proportions	Pupil size
Dominant percept	*F*_(1,9)_ = 4.47 *p* = 0.06 logBF = 0.19	*F*_(1,9)_ = 18.40* p = 0.002 logBF = 13.99
		
Contrast increment	*F*_(4,9)_ = 4.23* p = 0.007 logB*F* = −0.44	*F*_(4,9)_ = 1.16 *p* = 0.34 logBF = −1.30
		
Dominant percept × contrast increment	*F*_(4,9)_ = 39.75* p < 0.001 logBF = 12.36	*F*_(4,9)_ = 14.11* p < 0.001 logBF = 3.38

Two-way ANOVA for contrast enhancement results, with factors: dominant percept (white/black disk) and contrast increment (0%, 25%, 50%, 100%, 150%). Greenhouse–Geisser corrected values. * for *p* < 0.05 or lower.

However, in line with the results of the main experiment, the effects of attention cueing on perceptual dominance and pupil modulations were very different. While cueing the white disk modulated perceptual dominance in favor of the white stimulus, it did not modulate the relative pupil response (Fig. 5*A*,*B*, rightmost point; Table 3), replicating our main experiment results. These observations were further supported by directly comparing the cueing condition with the contrast condition that better mimicked the effect of attention on dominance proportions: the 100% contrast increase (Table 3), in line with the estimates reported in Chong et al. (2005). Cueing the white disk and increasing its contrast by 100%, despite producing comparable behavioral results, elicited very different pupil responses. While the 100% contrast increase elicited significantly stronger pupil modulations than in the equal contrast (no cue) condition, pupil modulations when the white disk was cued were indistinguishable from those in the no cue condition (Fig. 5*C*).

**Table 3:** The effects of cueing on proportions and pupil size in the control experiment

	Proportions	Pupil size
Modulation index: no cue vs. cueing	*t*_(9)_ = 5.66* p < 0.001 logBF = 2.04	*t*_(9)_ = 0.66 *p* = 0.52 logBF = −0.43
		
25% contrast increment vs cueing	*t*_(9)_ = 5.46* p < 0.001 logBF = 1.95	*t*_(9)_ = 0.41 *p* = 0.69 logBF = −0.48
		
50% contrast increment vs cueing	*t*_(9)_ = 2.43* p = 0.04 logBF = 0.33	*t*_(9)_ = 0.52 *p* = 0.61 logBF = −0.45
		
100% contrast increment vs cueing	*t*_(9)_ = 0.77 *p* = 0.46 logB*F* = −0.40	*t*_(9)_ = 3.29* p = 0.01 logBF = 0.81
		
150% contrast increment vs cueing	*t*_(9)_ = 3.83* p = 0.004 logBF = 1.11	*t*_(9)_ = 4.73* p = 0.001 logBF = 1.58

Modulation indices from the control experiment: comparison of attention cueing versus no cue (first row) and attention cueing versus contrast enhancement by 25%, 50%, 100%, and 150%. * for *p* < 0.05 or lower.

As a confirmatory analysis, we also checked the cross-correlation between the rate of pupil size change and perceptual reports (see Materials and Methods). The peak cross-correlation was significantly larger than zero in no-cue conditions (*t*_(37)_ = 5.41, *p* < 0.001, logBF = 3.70), further confirming that pupil modulations reliably tracked perceptual alternations; and it was higher when the white stimulus contrast was doubled (control experiment +100% contrast, *t*_(9)_ = 2.32, *p* = 0.04, logBF = 0.28), indicating that it is indicative of stimulus strength. However, the peak cross-correlation was indistinguishable across cueing conditions (white cued vs no cue: *t*_(37)_ = 1.23, *p* = 0.22, logBF = –0.45; *t*_(9)_ = 0.83, *p* = 0.42, logBF = –0.39, in the main and the control experiment, respectively), confirming that cueing did not alter the reliability of pupil modulations.

### Analysis of mixed percepts

All the analyses presented to this point are focused on exclusive dominance phases. In this section, we consider perceptual and pupillary reports for the third perceptual state that participants had the option to report: mixed percepts, defined as anything but the exclusive dominance of a white or black disk percept.

Previous work (Brascamp et al., 2006; Dieter et al., 2017) highlighted the importance of quantifying the fraction of return transitions, where mixed percepts are interposed between two periods of dominance of the same percept (e.g., black disk percept, followed by mixed percept, followed by another black disk percept). In no-cueing conditions, these return transitions represented a small fraction of all transitions (∼15%) for binocular rivalry and a greater fraction for interocular grouping rivalry (∼40%). Attention cueing dramatically affected the distribution of these transitions, as the proportion of black-mixed-black transitions increased when cueing black and white-mixed-white transitions increased when cueing white. This resulted in a significant interaction between factors “dominance percept after a return transition” and “cued percept” in a 2 × 2 ANOVA for both binocular rivalry (*F*_(1,37)_ = 20.19, *p* < 0.001, logBF = 1.75) and interocular grouping rivalry (*F*_(1,37)_ = 12.91, *p* < 0.001, logBF = 2.94). This indicates that attention cueing also affected the frequency of dominance phases, besides modulating the duration of individual dominance phases.

**Figure 6.**
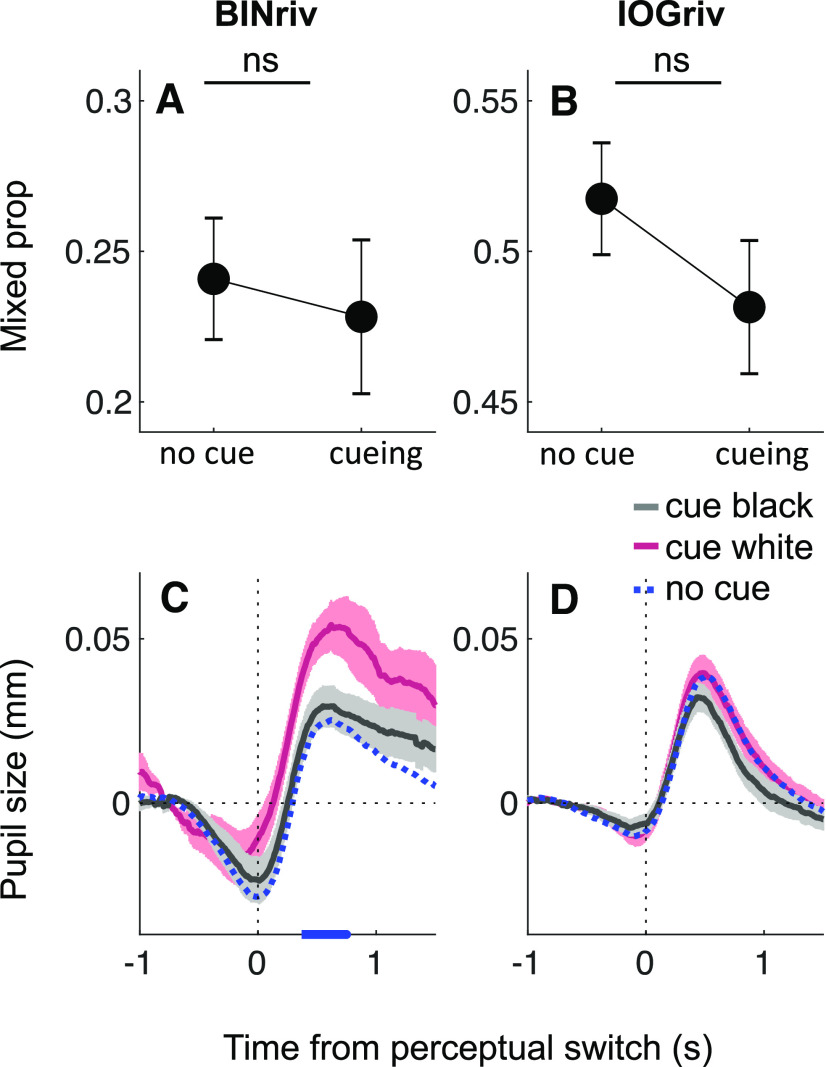
***A***,***B***, Proportion of mixed percepts in no cueing or cueing conditions (collapsed across white and black cued) for binocular rivalry (BINriv) and interocular grouping rivalry (IOGriv). Error bars report ±1 s.e. across participants. *ns*: not significant. ***C***,***D***, Pupil traces during mixed percepts in the three cueing conditions for binocular rivalry (BINriv) and interocular grouping rivalry (IOGriv). Shaded areas show ±1 s.e. across participants and blue marks on the *x*-axis highlight timepoints where pairwise comparisons between the white and black cueing conditions are significant (*p* < 0.05 FDR corrected).

Figure 6*A,B* quantifies the overall percentage of mixed percepts in binocular and interocular grouping rivalry. Attention cueing did not affect the proportion of mixed percepts in binocular rivalry, which averaged 0.24 ± 0.02 and 0.23 ± 0.02 for no cueing and cueing conditions (*t*_(37)_ = 0.80, *p* = 0.43, logBF = –0.63). In interocular grouping rivalry, the difference was also nonsignificant, but there was a trend toward reduced mixed reports in the cueing condition (0.52 ± 0.02 and 0.48 ± 0.02 for no cueing and cueing, respectively, *t*_(37)_ = 1.71, *p* = 0.10, logBF = –0.18). Based on this observation, we cannot exclude the possibility that attention cueing may have promoted interocular grouping.

Figure 6*C*,*D* shows pupil traces aligned to the onset of mixed percepts, separately for no cueing and the two attention cueing conditions; note that this is conceptually equivalent to analyzing data for exclusive dominance phases aligned to their offset, rather than the onset (same conventions as in Fig. 4*C*,*D*). A difference between cueing conditions is apparent for binocular rivalry, suggesting enhanced pupil dilation when the white percept was cued. No such effect is observed for interocular grouping rivalry. This difference between the two rivalry types may suggest that the cueing effect is specifically related to fusion percepts (gray disk percepts), which likely represented a minor percentage of mixed reports in interocular grouping rivalry (the largest majority being monocular half white, half black disk percepts). The relative pupil dilation may indicate that fusion events were perceived as gray disks of a darker shade during white cueing than during black cueing or no cueing. There are at least two reasons why this could happen. One possibility is that white cueing indeed enhanced the effective strength of the white stimulus, implying that the black stimulus strength had to reach a higher threshold before a white-dominance report switched to a mixed report. If this is the case, however, it is unclear why such difference in effective strength should not show in pupil traces during phases of exclusive white dominance (Figs. 3, 4). Another possibility is that cueing may have affected decision criteria so that fusion percepts reported as mixed were generally darker under white cueing (effectively prolonging exclusive white-dominance phases) than under black cueing conditions.

## Discussion

We used pupillometry to investigate the effects of endogenous attention on binocular rivalry and interocular grouping rivalry.

We confirmed that pupil size tracks perceptual oscillations during binocular rivalry, despite constant luminance stimulation, and we extended this observation to interocular grouping rivalry. This is consistent with the large body of work suggesting that the subcortical circuit generating the pupillary light response can be modulated by perceptual signals (Binda and Murray, 2015; Binda and Gamlin, 2017; Mathôt, 2018). Our finding that similar pupillary modulations accompany interocular grouping rivalry constrains the origin of the modulatory signals to visual cortical areas (they must hold a representation of stimulus brightness) with access to binocular information (they must be able to combine information from the two eyes).

We found that manipulating endogenous attention reliably affected perceptual alternations, enhancing dominance of the cued percept during binocular rivalry, in line with previous work (Meng and Tong, 2004; Mitchell et al., 2004; Chong et al., 2005; Hancock and Andrews, 2007; Paffen and Alais, 2011). To our knowledge, this is the first study manipulating attention in interocular grouping rivalry. We found that attention cueing had the same or slightly smaller effects on interocular grouping rivalry as on binocular rivalry. This suggests that eye-based and pattern-based competition are similarly permeable to endogenous attention; it also suggests that different degrees of attentional control (as observed, for example, comparing Necker cube vs binocular rivalry; Meng and Tong, 2004) may be related to differences in stimulus complexity rather than to the involvement of different levels (monocular vs binocular) of cortical processing.

Many have suggested that attention cueing acts by enhancing the perceptual strength of the cued signals (Carrasco, 2011). This is in line with evidence that focusing attention at a spatial location or feature enhances its representation in early visual cortex, as measured with EEG (Hillyard and Anllo-Vento, 1998; Di Russo et al., 2003; Wang et al., 2007; Kelly et al., 2008; Khoe et al., 2008; Mishra and Hillyard, 2009), fMRI (Saenz et al., 2002; Liu et al., 2005; Boynton, 2009; Pestilli et al., 2011), or indexed by enhanced pupillary response to light stimuli at the attended location (Binda and Murray, 2015). Transferring this knowledge to the context of rivalry, we expected that cueing attention to one of the rivaling percepts would enhance its effective strength and thereby increase its dominance. Using pupillometry, we intended to indirectly index this phenomenon. We established that the magnitude of pupil-size modulations accompanying rivalry is sensitive to effective stimulus strength as set by ocular dominance (control analysis of binocular rivalry data from the main experiment) or physical contrast changes (control experiment). On this basis, we predicted that attention cueing would have a similar effect as physical contrast enhancement, namely an amplification of pupil modulations. However, we obtained evidence against this prediction, as pupil responses during periods of exclusive dominance were reliably unaffected by attention cueing.

The simplest way to explain this negative finding is putting it down to insufficient sensitivity of the pupillometric measurements. However, our reliability analysis, Bayesian statistics and results from a control analysis and a control experiment all coherently speak against this possibility. We therefore speculate on a few logical alternatives.

During binocular rivalry, most of the time is spent in exclusive dominance, where competition between rivaling stimuli is resolved, leaving only one visible stimulus and no distracter. In these conditions, attention may be automatically driven to the dominant stimulus (Li et al., 2017), leaving little space for endogenous re-directing of attention. Although this is consistent with attention affecting early visual processing in markedly different ways at the onset of rivalry versus for nonrivaling stimuli (Khoe et al., 2008; Mishra and Hillyard, 2009), the model by Li et al. (2017) does not explicitly account for the small but reliable effects of attention cueing on perceptual alternations during rivalry. To account for these, one possibility is assuming that attention cueing primarily affects rivalry when the competition between stimuli is unresolved, namely in the brief times marking transitions between exclusive dominance phases, when the depth of suppression decreases (Alais et al., 2010). This idea has been suggested previously and supported by the observation that exogenous cues are mostly effective when presented near the end of individual dominance periods (Dieter et al., 2015). In this scenario, we could reconcile our behavioral and pupillometry results by assuming that attention enhances the strength of cued percepts only in short intervals near perceptual switches, not during the entire dominance phases. This could be consistent with our observation that the only effects of cueing over pupil traces were observed in a brief interval during mixed percepts.

An alternative possibility is that attention cueing affects rivalry dynamics by acting on a stimulus representation that is not represented in pupil dynamics. Available evidence is consistent with pupil size integrating a cortical representation of stimulus brightness (e.g., one that oscillates, tracking rivalry dynamics), but we lack direct knowledge on the level at which such visual representation is generated and fed into the pupil control circuit (Binda and Gamlin, 2017). On the other hand, evidence indicates that rivalrous perception is orchestrated by the interplay of fronto-parietal and occipital regions, which participate in different degrees depending on details of the stimulus and task (Logothetis et al., 1996; Sterzer and Kleinschmidt, 2007; Sterzer et al., 2009). It is possible, then, that attention affects competition after the stage where visual representations are fed to pupil control, whether this needs to be a decisional stage or still a sensory representation cannot be determined based on the available research.

This is not the first case where we find that the pupillary responses are independent of physical luminance and yet inconsistent with perceptual judgments (Benedetto and Binda, 2016; Turi et al., 2018; Pome et al., 2020; Tortelli et al., 2021a,b). These inconsistencies were generally explained by calling decisional factors into the picture, as these may bias or add variability to perceptual reports while leaving pupil size unaffected (Tortelli et al., 2021b). That contextual factors other than physical luminance affect pupil size and perception similarly but independently, if it proves recurrent and reliable across paradigms, might call for an updated model of pupil control. It might suggest that separate processing pathways support perception and pupil control, in analogy (or perhaps in overlap) with the separate pathways supporting vision for perception and vision for action (Goodale and Milner, 1992).

In conclusion, we find that pupil size oscillates in phase with perceptual oscillations during binocular and interocular grouping rivalry, implying cortical control. Despite this, and despite the reliable effects of attention cueing on behavioral reports, pupil size during periods of exclusive dominance does not show any modulation with attention. This introduces new constraints for models of attention in rivalry and pupil control: either attention cueing affects perception without enhancing the dominant percept, or we hold multiple representations of the dominant percept that independently regulate behavioral reports and pupil size and are differentially affected by attention cueing.

10.1523/ENEURO.0497-21.2022.t1-1 Extended Data Table 1-1 Three-way ANOVA for attention cueing results. Three-way ANOVA for attention cueing results, with factors: dominant percept (white/black disk), cueing (white/black cued), rivalry type (binocular/interocular grouping rivalry). We confirmed our results on cueing with a three-way ANOVA entered with the average pupil size in the interval [–0.5:1] s (the same interval used for Table 1 in the main text) but now skipping the baseline correction step (first column) or subtracting a baseline computed as the average pupil size in the [–5:5] s interval around perceptual switch (second column). In both cases, we confirm the main effect of dominant percept type and the absence of any reliable effect of attention cueing, suggesting that our results are not limited to the specific window we used to compute the baseline pupil size. Download Table 1-1, DOC file.

## Synthesis

Reviewing Editor: Alexander Soutschek, Ludwig-Maximilians-Universitat Munchen Decisions are customarily a result of the Reviewing Editor and the peer reviewers coming together and discussing their recommendations until a consensus is reached. When revisions are invited, a fact-based synthesis statement explaining their decision and outlining what is needed to prepare a revision will be listed below. The following reviewer(s) agreed to reveal their identity: R Ebitz. Note: If this manuscript was transferred from JNeurosci and a decision was made to accept the manuscript without peer review, a brief statement to this effect will instead be what is listed below.

The manuscript was reviewed by two reviewers, one of them had already reviewed the manuscript for JNS. The reviewers agree that the revisions substantially improved the quality of the manuscript, but several (mostly minor) issues remain that need to be addressed in a further revision.

The reviewers suggest two improvements to the analysis to ensure that the lacking effect is not caused by some crucial analysis steps. The analysis and signal-to-noise ratio (SNR) can be improved by removing a more complex trend in pupil size across 3min trials (remove a low-pass fit or apply a high-pass filter) and by subtracting baseline in a more structural (and less arbitrary) way per perceptual switch (see the comments below for details).

The authors should also discuss the possibility that their effects might change if the effect of black-versus-white cueing is performed in combination with a higher contrast (150%) between larger (>5 degrees) disks. By increasing the SNR of the pupillary effect, the authors would have designed the ultimate condition to determine whether endogenous cueing of attention in binocular rivalry really does not affect pupillary dynamic, meaning that the pupil does not supposedly reflect dominance strength.

Comments per line

Line 93-94: The authors might want to cite work by Josh Wang and David Munoz. They have made explicit claims about stimulus salience and pupil orienting response, and how these mechanisms interact.

Line 130: Was the screen linearized by removing the gamma-like relation between input values and screen luminance output values, e.g. through changes in the color look-up table? The reported luminance values suggest this was the case but the reviewers could not find an explicit note about this.

Line 137-138: For unclear reasons, the stimuli were relatively small and dark (white stimulus: 28 cd/m2). Choosing such a weak contrast between the dark and white stimulus may have potentially reduced effects on the pupillary changes, perhaps also explaining the absence of an effect of perceptual cueing. The authors should elaborate on this.

Line 140: It is Michelson contrast, not Michaelson.

Line 162: keypress-errors may not be the only possibility. Very fast perceptual switches or return-transitions may explain this as well.

Figure 1. The schematic visualization of mixed perception is a bit unrealistic. The pupil trace in Figure C looks like a (simulated) drawn rather than a real pupil trace.

Line 181: Did the authors simulate fast traveling waves (sweeps) between percepts with half-transparent gradients between perceptual borders? It should also be made clear why this condition was added in the methods.

Line 213: Removing a linear trend is a rather crude way of removing slow fluctuations in pupil size that are unrelated to the dynamics in brightness during rivalry. The reliability of the reported effects can be improved by removing a more complex trend: subtract a high-pass Butterworth filtered fit with a threshold frequency of 0.25-0.5Hz.

Line 237: It is unclear why the period of 1 to .5secs before a perceptual switch was chosen for the baseline-subtraction calculation. This builds in an arbitrariness in the analysis, where the chosen period possibly affects how the data looks. For instance, the difference between black-white may look totally different if a period of -250:+250secs is used for the baseline subtraction. The authors should somehow show that the effects generalize when using other baseline periods (show effects across a moving window for the baseline) or perhaps it is better to use the average across an entire switch epoch rather than a pre-switch baseline period.

Line 317-320: move the rational behind the replay/simulation condition to the methods where it belongs. The simulation remains a limitation of the study and needs to be discussed later in the paper.

Line 322-334: the authors use up a lot of text to discuss a non-significant effect. The reviewers wonder if the effect becomes significant after the different implementation of removing trends and baseline-correction. If it remains a trend, the authors should remove the speculative discussion (mentioning a trend is ok but then do not value it too much).

Line 445: “not only did we lack evidence of an”. Please check grammar.

Figure 4D: this is figure is confusing because the y-axis displays attentional cueing/modulation indices (AMI) but it is unclear whether the authors cued the white versus black stimulus also in high contrast conditions. If not, then the y-axis is incorrect. It is a pity that the authors didn’t apply the cueing paradigm in the 150% condition to calculate the AMI.

Line 566-570: The authors should discuss their findings in the light of previous studies that tested for the same pupil effect with very similar designs. The reviewers believe that Manfred Fahle & Wolfgang Einhauser were the first to use the pupillary luminance-tagging paradigm in binocular rivalry. Did the results differ in effect size or pupil patterns across time from these previous studies? A more thorough reflection on previous literature would make this paper more comprehensive.

The reviewers also wonder whether the difference in pupil responses between simulated and “normal” rivalry is something that the authors expected based on previous literature. Please discuss.

Line 605:621: The authors provide feasible explanations for the null-result. The reviewers wonder whether they considered the possibility that attentional switches between bright and dark during perceptual switches account for the rather subtle changes in pupil size (∼0.05mm), and that the attentional cueing would be another weak attentional effect on top of an already present though weak attentional effect? In the context of signal-to-noise ratios and ceiling effect, wouldn’t it be expected that no effect is observed? Again, increasing the contrast between the rivalling stimuli together with a cueing task would enhance SNR substantially. The authors should either test this or at least discuss this possibility.
