# Supplementary figures and images for: Attention Cueing in Rivalry: Insights from Pupillometry
Source: eNeuro. 2022 Jun 22;9(3):ENEURO.0497-21.2022. doi: 10.1523/ENEURO.0497-21.2022 (PMC9224166; doi:10.1523/ENEURO.0497-21.2022)

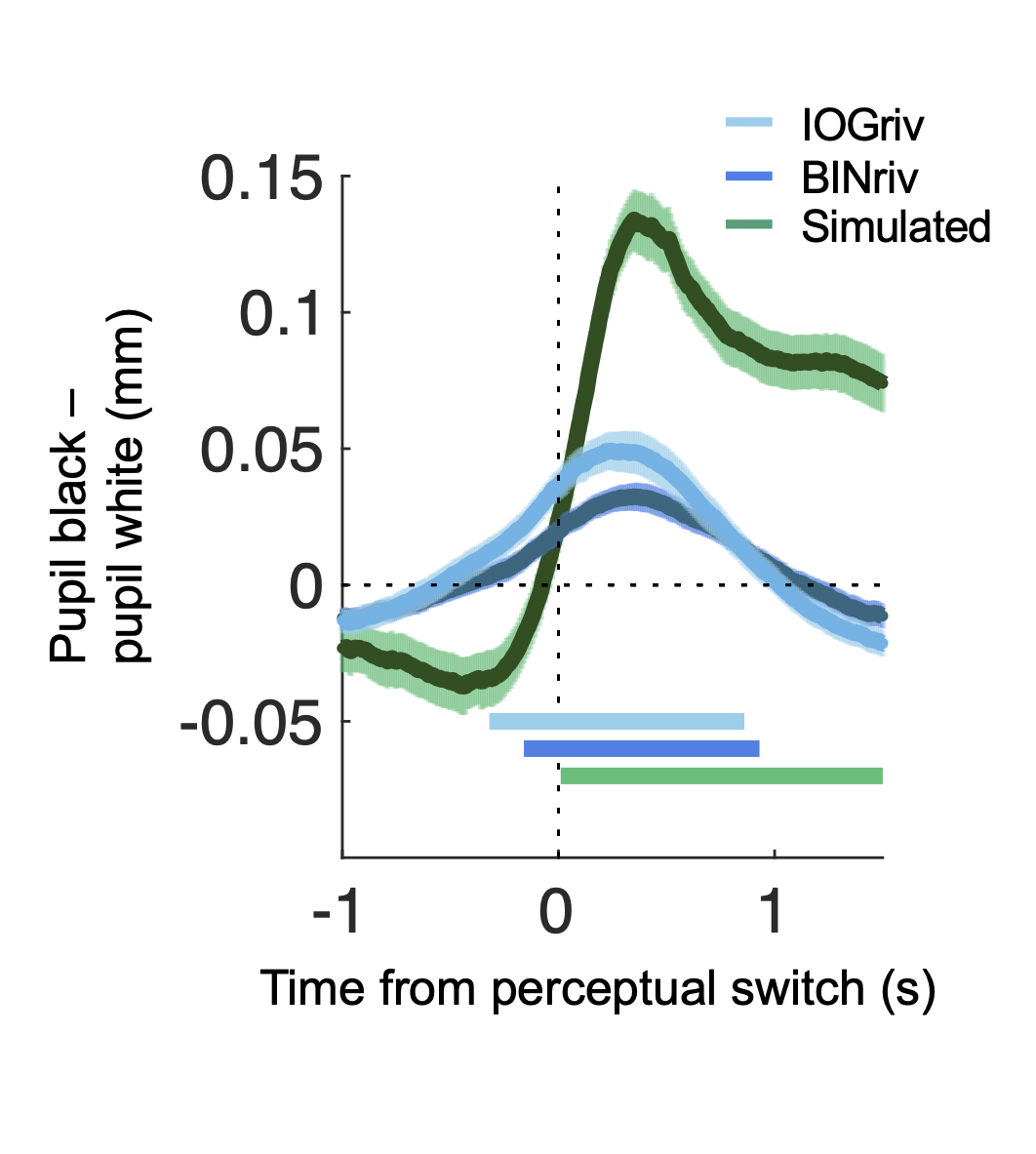

Supplement: Extended Data Figure 2-1 — Pupil size modulations in rivalry and simulation conditions. Time course of the difference between pupil size during black and white percepts, computed in individual participants, and then averaged for binocular rivalry (dark blue curve), interocular grouping rivalry (light blue curve) and simulation (green curve). In all panels, shadings report mean ±1 SE across participants and the blue marks on the x-axis highlight time points where each trace is significantly different from 0 (one tailed t test, p < 0.05 FDR corrected). Pupil modulations during binocular and interocular grouping rivalry averaged, respectively, 39.14 ± 8.15% (mean ± 1 SE across participants) and 43.80 ± 10.26% of the pupil size modulations observed during simulated rivalry, as previously reported by Binda and colleagues (Binda et al.,2013) in a different spatial-attention task. Moreover, they consistently started before the perceptual switch, and this was more pronounced in the rivalry conditions than in the simulated rivalry (significant pupil difference started 160 ms before the switch in binocular and 320 ms in interocular grouping rivalry, compared with almost no latency for simulated rivalry). This finding, in line with Fahle et al. (2011) and Naber et al. (2011), may reflect the graded nature of rivalry transitions, which may delay change detection in rivalry compared with the sharp transitions used in the simulated rivalry condition. Download Figure 2-1, TIF file. [file enu-eN-NWR-0497-21-s02.tif]

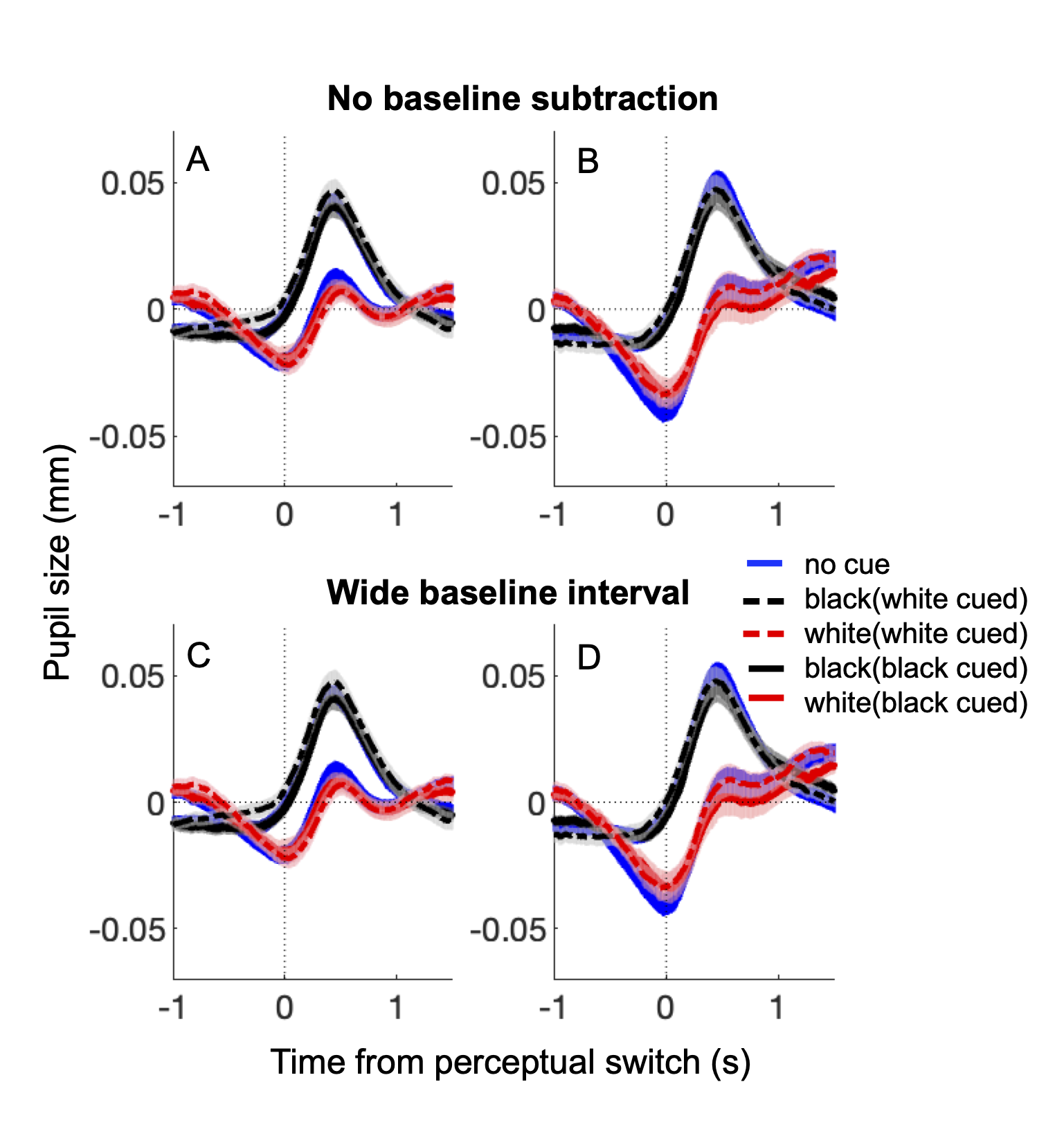

Supplement: Extended Data Figure 4-1 — Pupil modulations track perceptual alternations comparably across cueing conditions irrespectively of whether and how pupil traces are baseline corrected. Pupil size traces aligned to perceptual switches towards exclusive dominance of a white disk or a black disk percept and averaged across phases, separately for binocular rivalry (A) and interocular grouping rivalry (B) and separately for the two cueing conditions: cueing the white percept (dashed lines) or the black percept (continuous lines). Shading report ±1 SE across participants. These traces are computed without subtracting any baseline correction (A, B) and after subtracting a baseline computed as average pupil size in the [–5:5] s interval around perceptual switch (C, D). Note how the resulting traces are virtually indistinguishable: in both cases pupil size still allows to discriminate white and black percepts (red and black curves are clearly separated) but shows no effect of cueing (dashed and continuous lines are virtually superimposed, together with the blue traces). Coherently, we found that cueing did not affect preswitch pupil baseline used for the main figures, which was computed in the [–1:–0.5] s interval from the perceptual switch (main effect of cued percept: F(1,37) = 1.51, p = 0.23, logBF = –0.60; dominant percept × cued percept interaction: F(1,37) = 0.14, p = 0.71, logBF = –0.74). Download Figure 4-1, TIF file. [file enu-eN-NWR-0497-21-s03.tif]
