# Supplementary material for: Attention Cueing in Rivalry: Insights from Pupillometry
Source: eNeuro. 2022 Jun 22;9(3):ENEURO.0497-21.2022. doi: 10.1523/ENEURO.0497-21.2022 (PMC9224166; doi:10.1523/ENEURO.0497-21.2022)
Supplement: Extended Data Table 1-1 — Three-way ANOVA for attention cueing results. Three-way ANOVA for attention cueing results, with factors: dominant percept (white/black disk), cueing (white/black cued), rivalry type (binocular/interocular grouping rivalry). We confirmed our results on cueing with a three-way ANOVA entered with the average pupil size in the interval [–0.5:1] s (the same interval used for Table 1 in the main text) but now skipping the baseline correction step (first column) or subtracting a baseline computed as the average pupil size in the [–5:5] s interval around perceptual switch (second column). In both cases, we confirm the main effect of dominant percept type and the absence of any reliable effect of attention cueing, suggesting that our results are not limited to the specific window we used to compute the baseline pupil size. Download Table 1-1, DOC file. [file enu-eN-NWR-0497-21-s01.doc]

|  | No bsl sub | Bsl sub [-5 5] |
| --- | --- | --- |
| dominant percept | F_(1,37)_ = 40.09*  p < 0.001  logBF = 22.35 | F_(1,37)_ = 38.61*  p < 0.001  logBF = 21.73 |
| rivalry type | F_(1,37)_ = 2.17  p = 0.15  logBF = -0.52 | F_(1,37)_ = 2.12  p = 0.15  logBF -0.54 |
| cued percept | F_(1,37)_ = 2.58  p = 0.12  logBF = -0.61 | F_(1,37)_ = 2.72  p = 0.11  logBF = -0.57 |
| dominant percept  x rivalry type | F_(1,37)_ = 1.10  p = 0.30  logBF = -0.52 | F_(1,37)_ = 0.99  p = 0.32  logBF = -0.52 |
| dominant percept  x cued percept | F_(1,37)_ = 1.00  p = 0.32  logBF = -0.50 | F_(1,37)_ = 0.98  p = 0.33  logBF = -0.63 |
| rivalry type  x cued percept | F_(1,37)_ = 0.04  p = 0.85  logBF = -0.77 | F_(1,37)_ = 0.05  p = 0.82  logBF = -0.79 |
| dominant percept  x rivalry type  x cued percept | F_(1,37)_ = 1.67  p = 0.20  logBF = -0.51 | F_(1,37)_ = 1.77  p = 0.19  logBF = -0.49 |
